# Wood-like polymeric materials by ice templating

**DOI:** 10.1093/nsr/nwy139

**Published:** 2018-11-18

**Authors:** Sylvain Deville

**Affiliations:** Ceramic Synthesis and Functionalization Laboratory, National Center for Scientific Research, France

The ice-templating method, also called freeze-casting and directional freezing, is a powerful technique for processing nanomaterials, polymers and engineering materials into various porous and dense materials [1,2] that has received considerable attention. Ice templating is mostly used to fabricate macroporous materials, with elongated pores in the 5–50 ${\mu}$m size range. Because the underlying mechanisms—the segregation of matter by the growing crystals—are mostly physical and not chemical in nature, ice templating is a versatile process. All kinds of porous materials have been ice-templated, from polymers to ceramics, metals, and various types of composites [3]. Because of its versatility, a variety of functional properties and applications have been considered, such as multifunctional materials in sensors, tissue engineering or catalyst supports. Although the process is well understood today, industrial applications, with very few exceptions, still await. Despite low-temperature processes being very common in industries (think food engineering), little has been done on scaling up ice-templating processes. The mass production of porous, functional monoliths by ice templating is still a great challenge [3].

**Figure 1.**
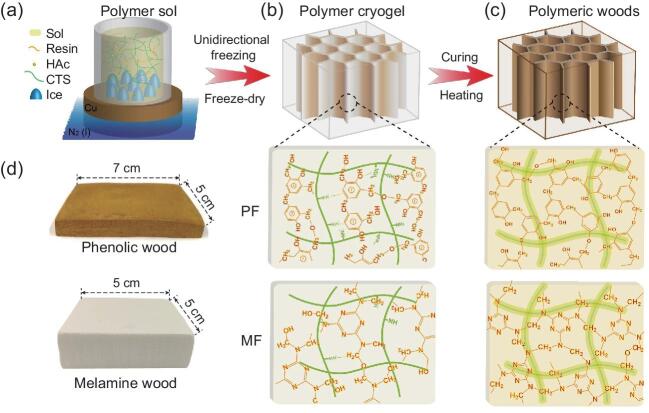
Schematic illustration showing the fabrication process of the artificial polymeric woods by using phenolic and melamine resins. (a) The polymer solution before freezing. (b) The obtained polymer cryogel after the freeze-drying process. (c) The final wood-like porous materials after thermocuring the resins. (d) Two kinds of typical artificial woods: phenolic wood and melamine wood. Figure reproduced from [4] (CC BY-NC 4.0 license).

A team led by Shu-Hong Yu at the University of Science and Technology of China have recently made progress in the mass production of a family of multifunctional porous polymers whose porous structures resemble wood, through ice templating and a subsequent curing process using traditional engineering resins (Fig. 1) [4]. The team chose thermoset phenolic and melamine resins as matrix materials, and several kinds of nanomaterials or molecules can be used as building blocks for the multifunctionality. The mechanical compressive performance of the porous materials (with compressive yield strengths of up to 45 MPa) is similar to or better than that of other cellular polymer or composite bulk materials of similar densities. These materials present an interesting combination of water and acid resistance (their mechanical properties decreased by less than 25% after immersion in water or sulfuric acid solution for 30 days), fire resistance (the materials self-extinguish quickly) and thermal insulation (as low as ∼21 mW m^−1^ K^−1^), and could thus be an alternative to the current thermal insulation and fire-resistant building materials [5]. Besides this useful combination of functional properties, this study is also a much-needed move towards the scaling-up of ice templating. The resin used here is less sensitive to the development of structural defects commonly encountered when trying to freeze and freeze dry particulate suspensions to shape pieces of larger dimensions, which is rarely attempted. More work will now be needed, in particular to increase the thickness of the pieces beyond 2 cm while maintaining a homogeneous structure and investigate whether biosourced materials could be used instead of the more traditional resins.
